# Suicidal Ideation, Cognitive Control, and Sleep in Veterans in a Residential Treatment Facility: A Pilot Study

**DOI:** 10.1111/sltb.70011

**Published:** 2025-04-01

**Authors:** Lily A. Brown, Kevin G. Lynch, Philip Gehrman, Lindiwe Mayinja, Danielle Farabaugh, Keith Bredemeier

**Affiliations:** ^1^ Department of Psychiatry University of Pennsylvania Philadelphia Pennsylvania USA; ^2^ Coatesville VA Medical Center Coatesville Pennsylvania USA; ^3^ Psychological and Brain Sciences University of Delaware Newark Delaware USA

**Keywords:** cognitive control, sleep disorders, suicide, veterans

## Abstract

**Introduction:**

Sleep disorder symptoms are associated with suicidal ideation, and wakefulness in the middle of the night is associated with an increased risk for suicide, potentially due to deficits in cognitive control. Little is known about daily‐level associations among sleep and suicide or about the role of cognitive control in these associations.

**Methods:**

Veterans (*n* = 32) with past‐month suicidal ideation in a residential treatment unit were assessed for 28 days using the suicide visual analogue scale (S‐VAS) to assess suicidal urges, a daily sleep diary, and self‐reported cognitive control.

**Results:**

Wakefulness in the middle of the night (1–4 a.m.) was associated with the most severe suicidal ideation. Nocturnal wakefulness and sleep quality were each associated with next‐day suicidal ideation intensity. Self‐reported cognitive control deficits were associated with more intense suicidal ideation. The association between nocturnal wakefulness and suicidal ideation was partially accounted for by self‐reported impulsive behavior (7% of variance).

**Conclusions:**

Veterans reported the highest suicidal ideation in the middle of the night. These findings suggest the importance of assessing sleep, suicidal ideation, and cognitive control among veterans and the need to consider nocturnal wakefulness as an indicator of heightened risk for suicide among veterans.

## 1 Introduction

Suicide rates have been increasing in the last two decades (Centers for Disease Control and Prevention 2014; Garnett and Curtin 2024); yet there are few reliable predictors of who is going to die by suicide (Franklin et al. 2017). Of all suicide deaths in the United States, 22.2% are accounted for by veterans, with approximately 20 veteran suicides daily (U.S. Department of Veterans Affairs 2023). In addition, the monthly rate of veteran non‐fatal suicidal events, including episodes of severe suicidal ideation or suicide attempts, has increased since 2007 (U.S. Department of Veterans Affairs 2023). Among veterans recently discharged from residential facilities, 63% of suicide attempts occurred in the first 90 days after discharge (Bishop et al. 2019). Given the elevated suicide risk among veterans, there is an urgent need to improve the ability to predict and intervene upon suicide risk in veterans in real time. Understanding changes in suicidal ideation might be helpful in this regard, given that suicidal ideation is associated with increased risk for future death by suicide (Louzon et al. 2016; Wenzel et al. 2011).

Cross‐sectional studies have demonstrated that suicide death is more likely to occur when individuals are awake in the middle of the night, with the greatest risk between 2 and 3 a.m., in civilians (Perlis, Grandner, Brown, et al. 2016) and veterans (McCarthy et al. 2019). Among veterans living in a residential facility, the presence of insomnia symptoms was more common among those who made a suicide attempt following discharge than among those who did not (62% vs. 11%; Bishop et al. 2019). Therefore, sleep disorder symptoms may be important to intensively assess among veterans who are living in residential facilities and are at heightened risk for suicide. The nocturnal wakefulness–suicide death connection is especially concerning for veterans with PTSD. Approximately 50% of veterans report sleep disorder symptoms (Mustafa et al. 2005), and 70%–90% of individuals with post‐traumatic stress disorder (PTSD) report sleep disturbances (Pace‐Schott et al. 2015). Up to 27% of veterans with PTSD report lifetime suicidal ideation (Straus et al. 2019). However, no research has investigated how changes in nocturnal wakefulness contribute to suicidal ideation in veterans with PTSD.

One critical factor influencing the nocturnal wakefulness–suicide risk association may be decreased cognitive control (Perlis, Grandner, Chakravorty, et al. 2016). Cognitive control is measured by one's ability to resist impulses and stay focused on a task, which is needed to modulate emotional systems in goal‐directed behavior when habitual responses are not sufficient for the current context. Several reviews have shown that impairment on measures of cognitive control is reliably associated with suicidal ideation (Keilp et al. 2008; Westheide et al. 2008), attempts (Bredemeier and Miller 2015; Richard‐Devantoy et al. 2014), and with progression from suicidal ideation to action (Saffer and Klonsky 2018). Existing research on cognitive control and suicidality has used cross‐sectional or retrospective designs; thus, while cognitive control deficits are thought to be a risk factor for suicidal thinking and behavior (Bredemeier and Miller 2015), this has not been tested using prospective studies.

One approach to investigate the relationships among nocturnal wakefulness, cognitive control, and suicide risk in real time is through the use of ecological momentary assessment (EMA). EMA provides real‐time data on cognition, affect, and behavior through smartphone notifications and is feasible and acceptable in samples with suicidal ideation (Husky et al. 2017). Several studies have used this method to examine changes in suicidal thoughts and behavior, demonstrating that suicidal ideation varies significantly from one rating to the next (Husky et al. 2017; Kleiman and Nock 2018). However, these studies did not attempt to associate nocturnal wakefulness, cognitive control, and suicide risk over time.

The first aim of this project was to evaluate associations between nocturnal wakefulness and suicidal ideation prospectively in veterans with PTSD in residential treatment. In residential treatment in the VA, a veteran is typically not in an imminent suicidal crisis at the time of admission, though they may have recently been discharged from the hospital following a suicidal crisis. We hypothesized that a longer duration of nocturnal wakefulness would be associated with higher levels of next‐day suicidal ideation. We also explored whether time to fall asleep and subjective sleep quality were each associated with next‐day suicidal ideation and whether participants reported that suicidal ideation was more likely at particular times. Consistent with our prior research on the timing of suicidal ideation (Brown et al. 2022) and data on the timing of suicide mortality (McCarthy et al. 2019; Perlis, Grandner, Brown, et al. 2016), we hypothesized that suicidal ideation would peak in the middle of the night between midnight and 3 a.m. The second aim was to evaluate associations between nocturnal wakefulness and next‐day cognitive control. We hypothesized that a longer duration of nocturnal wakefulness would be associated with higher levels of next‐day suicidal ideation. The third aim was to evaluate associations between cognitive control and suicidal ideation prospectively in veterans in residential treatment. We hypothesized that reduced cognitive control, assessed using EMA, would be associated with higher subsequent suicidal ideation. The final aim was to evaluate evidence for cognitive control as a mediator of the association between nocturnal wakefulness and suicidal ideation prospectively in veterans with PTSD. We hypothesized that the association between nocturnal wakefulness and suicidal ideation would be partially mediated by reduced cognitive control performance.

## 2 Materials and Methods

### 2.1 Participants

Veterans ages 18–75 with posttraumatic stress disorder (*N* = 32) were recruited from the residential PTSD treatment unit at the Coatesville Veterans Affairs Medical Center. Inclusion criteria were a current diagnosis of PTSD (verified through health record and provided by a licensed mental health clinician who was the chief clinical care coordinator for the veteran), past month suicidal ideation, past month insomnia as indicated by a score ≥ 15 on the Insomnia Severity Index (ISI), at least 18 years old, fluency in English, possession of a smartphone device, and ability to provide consent. Exclusion criteria were psychotic disorders (assessed using the Diagnostic Interview for Anxiety, Mood, and OCD and Related Neuropsychiatric Disorders [DIAMOND]; Tolin et al. 2018), current pregnancy, and uncontrolled respiratory, neurological, or cardiovascular diseases, if determined by the team to significantly impact the ability to participate. Demographic information is presented in Table 1. Our consort table reflecting recruitment information is included in Figure S1.

**TABLE 1:** Demographic and baseline clinical characteristics.

Demographic	Mean	SD
Age (years)	44.19	11.01
PTSD checklist	61.16	10.49
Beck Depression Inventory	28.4	12.37
Insomnia Severity Index	21.74	3.61

Demographic	Category	Frequency	%
Gender	Female	8	25
	Male	24	75
Ethnicity	Hispanic	6	18.75
	Non‐Hispanic	26	81.25
Race	White	25	78.13
	Black or African American	6	18.75
	American Indian or Alaska Native and White	1	3.13
Relationship status	Single	6	18.75
	Married	7	21.875
	Separated	3	9.375
	Married and separated	1	3.125
	Divorced	11	34.375
	Living with	2	6.25
	In a relationship	1	3.125
	None of the above	1	3.125
Number of children/stepchildren	0	6	18.75
	1	6	18.75
	2	7	21.875
	3+	11	34.375
	No response	2	6.25
Highest degree earned	Associates degree	4	12.5
	Bachelor's degree	7	21.875
	Doctorate degree	2	6.25
	High school or GED	6	18.75
	Master's degree	4	12.5
	Some college	9	28.125
Employment status	Full time	10	31.25
	Part time	3	9.375
	Retired	3	9.375
	Disabled	12	37.5
	Student	1	3.125
	Unemployed	3	9.375
Pre‐tax income	$0–$5000	2	6.25
	$10,001–$20,000	4	12.5
	$100,001+	2	6.25
	$20,001–$30,000	2	6.25
	$30,001–$50,000	11	34.375
	$50,001–$100,000	9	28.125
	Missing	2	6.25

### 2.2 Measures

#### 2.2.1 Suicide‐Visual Analogue Scale

Three suicide‐visual analogue scale (S‐VAS) probes were sent at random times in the morning (between 7 a.m. and 12 p.m.), afternoon (between 12 and 5 p.m.), and evening (between 5 and 11 p.m.). These three random probes were sent with the following instruction: “Show how extreme you are experiencing each of the feelings and moods right now, at the current moment: urge to kill myself,” with scores ranging from 0 to 100. In addition, on the morning sleep diary participants were asked, “Show how extreme you experienced this feeling/mood at any point last night: urge to kill myself,” and on the nighttime sleep diary participants were asked, “Show how extreme you experienced this feeling/mood at any point today: urge to kill myself.” The validity of the S‐VAS measure for quantifying suicide risk is well supported (Bryan 2019). Among a clinical sample of military personnel experiencing suicidal ideation, the S‐VAS showed good predictive validity, as baseline S‐VAS scores significantly predicted suicide attempts during the follow‐up assessment period (1, 3, and 6 months post‐intervention). The S‐VAS's responsiveness was also supported, as it is a reliable measure of two dimensions of clinical response, having a large effect size in measuring change in current suicidal ideation from baseline through 1 month post‐intervention and a moderate effect size in measuring early remission of ideation. Bryan's (2019) findings support the S‐VAS's specificity to measuring recent suicidal thinking and internal consistency with the measure's intended design to quantify proximal rather than distal suicide risk. The S‐VAS is a valid and useful measure for monitoring changes in suicide risk over brief periods of time and is thus a practical method for assessing fluctuations in suicidal ideation among veterans in the current study. The maximum S‐VAS was defined as the maximum of the S‐VAS assessed in the morning (7 a.m.–12 p.m.), afternoon (12–5 p.m.), or evening (5–11 p.m.).

#### 2.2.2 EMA Assessment of Cognitive Control

Participants completed daily Likert‐scale EMA probes of their behaviors related to cognitive control, including off‐task behaviors (e.g., mind‐wandering, browsing the internet), impulsive behaviors (e.g., aggression, overeating), and items derived from the Behavior Rating Inventory of Executive Function inhibit scale (e.g., “I was impulsive”; Gioia et al. 2000). More specifically, these EMA probes consisted of 3 items rated on a 5‐point scale ranging from 1 (strongly agree) to 5 (strongly disagree) assessing behavior and focus of attention; 8 items rated on a 5‐point scale ranging from 1 (not at all) to 5 (always) assessing engagement in off‐task, impulsive, or risk‐taking behaviors; and 6 items rated on a 3‐point scale (1 = never a problem; 2 = sometimes a problem; 3 = often a problem) assessing impulsivity. These questions were administered once during the day at a random time between 7 a.m. and 12 p.m. These probes asked about the frequency of behaviors over the past 24 h. Internal consistencies of these EMA cognitive control scales were acceptable (BRIEF inhibit scale: *α* = 0.93; impulsive behavior: *α* = 0.59; off‐task behavior: *α* = 0.71).

#### 2.2.3 Nocturnal Wakefulness

Participants completed a sleep diary between 6 a.m. and 12 p.m. each day for 28 days. As part of the morning sleep diary (Carney et al. 2012), participants were asked, “How long did it take you to fall asleep in minutes?” which was converted into hours for analyses, and “How many times did you wake up, not counting your final awakening?” and then “In total, how long did these awakenings last in minutes?” In addition, participants were asked, “How would you rate the quality of your sleep?” on a scale ranging from 0 (very poor) to 4 (very good).

### 2.3 Procedure

The study was approved by the Institutional Review Boards of the Coatesville VA Medical Center (1599126) and the University of Pennsylvania. Following the completion of informed consent, participants completed an eligibility assessment and then downloaded the Metricwire mobile application to their phones to begin receiving EMAs for 28 days.

### 2.4 Data Analysis

Our analyses used linear mixed effects models (Fitzmaurice et al. 2011). We included nocturnal wakefulness from the night before (time awake in the middle of the night) as a time‐varying covariate in a model predicting next day maximum suicidal ideation (using the S‐VAS, and taking the maximum score across the morning, afternoon, and evening random prompts); we expressed the wakefulness variable in hours rather than minutes. To address large values of positive skewness in both the predictor and response, and to improve the linearity of response, we square‐root transformed the nocturnal wakefulness predictor and maximum S‐VAS response prior to analyses. For the covariance structure in these models, we included a person‐level random intercept to accommodate within‐person correlations. After examination of the individual trajectories of response across days, we modeled within‐person time trends using linear trends. To allow for possible variation in the effect of nocturnal wakefulness across different periods of the following day, we examined the nocturnal wakefulness by period interaction, which is reported in supplemental analyses. We then repeated these analyses using the time to fall asleep variable and the subjective sleep quality variable from the previous night, replacing the nocturnal wakefulness variable.

We followed procedures from Perlis, Grandner, Chakravorty, et al. (2016), and Brown et al. (2022) to evaluate whether there were differences in the timing of when participants reported that their suicidal ideation was highest on their morning and nighttime sleep diary. During the morning sleep diary, participants were asked to reflect on their highest S‐VAS score at any point during the prior night and, when providing a non‐zero response, the timing of the highest S‐VAS. During the nighttime sleep diary, participants were asked to reflect on their highest S‐VAS score at any point that day and, when providing a non‐zero response, the timing of the highest S‐VAS. The frequencies of endorsing non‐zero responses were binned across each hour and are reported separately for the morning and nighttime sleep diary. These analyses account for the likelihood that participants would be awake at a given hour (using the ATUS Home, n.d.; Perlis, Grandner, Brown, et al. 2016) and calculate a standardized incidence ratio of the observed frequency of maximum suicide urges compared to the expected frequency of maximum suicide urges. When the 95% confidence interval for the standardized incidence ratio does not include zero, the ratio is statistically significant.

We addressed associations between nocturnal wakefulness and cognitive control using similar linear mixed effects models to those described above, with next‐day cognitive control EMA responses as the outcomes and previous night sleep indicators as explanatory variables. Next, we extended analyses of S‐VAS response, using cognitive control EMA data as the predictor variable, with analogous specifications for the fixed and random effects. In these analyses, our response variable is the maximum of the S‐VAS reports for the 12 to 5 p.m. and 5 to 11 p.m. periods, as opposed to in prior analyses where we also considered the S‐VAS from 7 a.m. to 12 p.m.

For mediation analyses, we used mixed effects models, similar to those described for Aim 2, with maximum S‐VAS post 12 p.m. as the response, with nocturnal wakefulness as the primary predictor variable, and the cognitive control measures included as (separate) mediators; the models included a variable for study day, and a person‐level random intercept accounted for within‐participant correlations. Models that included random effects to allow the paths from predictor to mediator and from mediator to response to vary across participants exhibited convergence problems and numerical instabilities (non‐positive‐definite Hessians), so we focused on a fixed‐effects approach, based on the method of Baron and Kenny (1986), noting that this approach can be underpowered: to assess mediation, we compared the regression estimates and their associated *p* values for the total (Aim 1) effect of nocturnal wakefulness and the direct effects (Aim 3) after the cognitive measures were included in the model (MacKinnon 2008). In all comparisons, we use *α* = 0.05 as the threshold for significance.

## 3 Results

The timing of self‐reported highest suicidal urges on the morning sleep diary, evening sleep diary, and combined across both sleep diaries is reported in Tables S1–S3. For the morning sleep diary, the 1 a.m., 2 a.m., 3 a.m., 4 a.m., 10 p.m., and 11 p.m. timepoints were all endorsed at rates significantly higher than expected. For the nighttime sleep diary, only 4 a.m. and 7 p.m. were endorsed at rates significantly higher than expected. Combining across both the morning and nighttime sleep diary, 1 a.m., 2 a.m., 3 a.m., 4 a.m., 10 p.m., and 11 p.m. were all endorsed at rates that were significantly higher than chance. Late morning until early evening (7 a.m.–6 p.m.) was endorsed at rates significantly lower than expected (SIR range: 0.28–0.83).

Results are based on 32 participants, contributing between 4 and 82 person‐day observations. The skewness values for the maximum S‐VAS response were 2.39, and skewness for raw maximum hours awake was 4.48; after square‐root transformation of the variables, the skewness values dropped to 0.96 and 1.07 for S‐VAS and hours awake, respectively.

In a mixed‐effects model that controlled for days since the start of the measurement period, the effect of nocturnal wakefulness on maximum daily S‐VAS was significant (*F*(1, 448) = 4.09, *p* = 0.044); a one standard deviation (SD) increase on the (square‐root transformed) nocturnal wakefulness variable was associated with a 0.073 (SE = 0.036, 95% CI = [0.002, 0.143]) SD increase on the transformed maximum S‐VAS response. In terms of within‐person correlations over time, the average daily Spearman correlation between nocturnal wakefulness and maximum S‐VAS ranged from −0.60 to 0.82, with a median value of 0.10, so the average effect of nocturnal wakefulness seems small and varies considerably across participants. Adjustment for baseline PTSD severity on the PTSD checklist (nocturnal wakefulness *p* = 0.031), baseline depression severity on the Beck Depression Inventory (*p* = 0.029), and baseline insomnia severity on the ISI (*p* = 0.036) yielded significant adjusted effects similar to the unadjusted effects. We also investigated the possibility of interaction effects of time period, which did not change the results reported in Supporting Information S1.

The relationship between time to fall asleep and next‐day S‐VAS was not significant (*F*(1, 448) = 0.52, *p* = 0.47); a one SD increase in the time to fall asleep variable was associated with a 0.046 (SE = 0.063, 95% CI [−0.078, 0.170]) SD increase in the transformed maximum S‐VAS response.

The average daily Spearman correlation between time to fall asleep and maximum S‐VAS ranged from −0.60 to 0.82, with a median value of 0.10. There was a significant association between subjective sleep quality (a five‐level ordinal variable) and next‐day S‐VAS that was significant (*F*(4, 444) = 2.81, *p* = 0.025); compared to a night when sleep quality was rated as “very good,” nights where sleep was rated as very poor (*p <* 0.01), poor (*p* < 0.01), and fair (*p* < 0.05) were associated with significantly higher next‐day S‐VAS. In contrast, nights that were rated good did not differ (*p* = 0.47) from those rated very good.

### 3.1 Cognitive Control and Suicidal Ideation

There was a positive association of daily self‐reported cognitive control, assessed using the EMA BRIEF inhibit scale assessed before 12 p.m., on suicidal ideation, assessed using the maximum S‐VAS assessed after 12 p.m. (*p* < 0.001), controlling for days since enrollment in the study. Participants who reported worse inhibition also reported higher S‐VAS. Likewise, there was a positive effect of cognitive control, assessed using the EMA impulsive behavior assessment assessed before 12 p.m., on suicidal ideation, assessed using the maximum S‐VAS assessed after 12 p.m. (*p* < 0.05), controlling for days since enrollment in the study, such that elevated impulsive behavior was associated with elevated S‐VAS. The effect of cognitive control, assessed using the EMA off‐task behavior assessment assessed before 12 p.m., on suicidal ideation, assessed using the maximum S‐VAS assessed after 12 p.m., was not significant (*p* = 0.769) controlling for days since enrollment in the study. The correlation between suicidal ideation and EMA BRIEF, off‐task behavior, and impulsive behavior scores was 0.32, 0.27, and 0.24, respectively, so the effects are in the small‐to‐medium range. For each of the three EMA responses, the effects were essentially the same when the models controlled for baseline psychopathology (PTSD severity, depression severity, insomnia severity).

### 3.2 Mediation Analysis

First, we note that the significant total effect (positive association) of nocturnal wakefulness noted above persisted here when we restricted to the 12–11 p.m. S‐VAS scores (beta coefficient = 0.78, SE = 0.25, *p* = 0.003 based on the person‐days in the EMA cognitive control sample). Effects of nocturnal wakefulness, assessed using the sleep diary, on cognitive control self‐report, assessed using the EMA impulsive behaviors (*p* = 0.40), EMA off‐task behaviors (*p* = 0.14), and EMA BRIEF (*p* = 0.40) before noon were all non‐significant. Furthermore, when the EMA impulsive behavior response was included as a mediator of the effect of nocturnal wakefulness on the maximum of the afternoon/evening S‐VAS scores, the direct effect of nocturnal wakefulness was 7% smaller than the total effect (beta coefficient = 0.72, SE = 0.25, *p* = 0.005), while the direct effects for EMA BRIEF (beta coefficient = 0.79, SE = 0.24, *p* = 0.002) and off‐task behavior (beta coefficient = 0.79, SE = 0.26, *p* = 0.003) scores were very similar to the total effect. The effects of the EMA variables in these models were similar to those reported above, with significant positive associations of d BRIEF (*p* < 0.001) and impulsive behavior (*p* = 0.10) on S‐VAS and a non‐significant effect for off‐task behavior (*p* = 0.80).

## 4 Discussion

In US veterans in a residential treatment setting, more nocturnal wakefulness and poorer sleep quality on a given night were associated with higher suicidal ideation on the subsequent day. Across all observations, participants reported that their suicidal ideation was highest in the middle of the night. Poorer cognitive control was also associated with worsened suicidal ideation later in the same day. In a mediation analysis, the effect of nocturnal wakefulness on next‐day suicidal ideation was accounted for to a small extent (7%) by self‐reported cognitive control, though the effect of nocturnal wakefulness continued to predict next‐day suicidal ideation strongly even with cognitive control in the model.

Several prior studies have demonstrated that sleep disorder symptoms are associated with higher levels of suicidal ideation (Bernert et al. 2015; Kivelä et al. 2024; Littlewood et al. 2019; Nadorff et al. 2013; Pigeon et al. 2016). Likewise, several prior studies have demonstrated an association between poorer cognitive control and increased risk for suicidal ideation (Keilp et al. 2008; Westheide et al. 2008) or attempts (Bredemeier and Miller 2015; Richard‐Devantoy et al. 2014). To our knowledge, this is the first study to demonstrate daily‐level associations among sleep disorder symptoms and suicidal ideation in a sample of veterans. This is an important contribution to the literature in that it demonstrates that the relationships between poor sleep and suicide risk are not just at the trait level but rather can change from day to day. These results are also important given that veterans are at elevated risk for suicide compared to the general population (U.S. Department of Veterans Affairs 2023).

This study builds on prior research in which individuals are at increased risk for suicide (McCarthy et al. 2019; Perlis, Grandner, Brown, et al. 2016) or suicidal ideation in the middle of the night (Brown et al. 2022). Pioneering researchers on this topic speculated that one possible explanation for this effect might be that the middle of the night is associated with deleterious outcomes on executive functioning ability (Perlis, Grandner, Chakravorty, et al. 2016). This study is the first of its kind to evaluate temporal associations between nocturnal wakefulness and subsequent cognitive control and suicidal ideation. We found that when a veteran spent more time awake on a given night, they had significantly worsened cognitive control the next day, which, in turn, was associated with significant elevations in suicidal ideation. Cognitive control deficits (specifically self‐reported impulsive behavior) partially accounted for the effect of nocturnal wakefulness on suicidal ideation, though this effect was small, accounting for only 7% of variance in the model.

In terms of limitations, the study was a pilot study and should be replicated with a larger sample size. This is especially important for a full evaluation of mediation effects, as our models might have been underpowered. In addition, when participants were awake in the middle of the night, we attempted to collect data on cognitive control and suicide risk in the middle of the night (to attempt to show worsened cognitive control performance in the middle of the night as a clear mechanism of this effect) by sending participants a silent notification, but adherence with those assessments was low and insufficient to analyze. This should be a direction for future research, potentially in a controlled sleep laboratory setting, to improve adherence. Participants were asked to use retrospective recall to reflect on the timing of elevated suicide risk in some assessments for the study (morning and evening sleep diary, but not for the random probed during the day in which participants provided real‐time ratings), and retrospective recall might have interfered with those assessments. Participants in this study were receiving treatment in a residential treatment unit, and it is possible that the pattern of results might not translate outside of this setting for veterans or civilians. It is also possible that receipt of treatment altered the pattern of results. However, when working with suicidal participants, it is best to encourage treatment engagement for ethical reasons, and thus naturalistic observation outside of treatment may prove difficult in future research.

This study has important clinical implications, particularly as it relates to the importance of assessing both sleep disorder symptoms and cognitive control deficits to understand changes in suicidal ideation for veterans. When working with veterans with suicidal ideation, it could be helpful to implement more comprehensive assessments of both sleep disorder symptoms and cognitive control. Based on our results, this could be addressed by assessing patients’ report on how much time they were awake in the middle of the night, their perception of sleep quality, and some items from the BRIEF. While these were assessed on a daily basis in our study, clinicians might benefit from brief weekly or even fortnightly assessments for their veterans to improve the conceptual model for each patient. Future research should evaluate the ideal frequency of assessment of all three constructs (sleep, cognitive control, and suicidal ideation) to prioritize efficiency for veterans and clinicians alike. Additionally, given that veterans consistently reported higher suicidal ideation in the middle of the night, clinicians might benefit from considering aspects of a Safety Planning Intervention (Stanley et al. 2018) or Crisis Response Plan (Bryan et al. 2017) that factor in intervention options that can be used in the middle of the night.

## 5 Conclusion

In summary, this study demonstrated daily‐level associations among sleep disorder symptoms, cognitive control, and subsequent suicidal ideation in veterans receiving treatment in a residential treatment unit. Veterans consistently reported that the middle of the night was a higher‐risk time. The association between sleep disorder symptoms and suicidal ideation was partially accounted for by cognitive control deficits (specifically impulsive behavior), though the association between nocturnal wakefulness and suicidal ideation was highly significant even after accounting for cognitive control. Future research should expand on these findings in a larger sample and explore deeper assessments of veterans who happen to be awake in the middle of the night to understand temporal mediators of increased risk during that window, including cognitive control deficits and other potential constructs of interest.

## Conflicts of Interest

The authors declare no conflicts of interest.

## Supporting information


Data S1.
