# Supplementary material for: Suicidal Ideation, Cognitive Control, and Sleep in Veterans in a Residential Treatment Facility: A Pilot Study
Source: Suicide Life Threat Behav. 2025 Apr 1;55(2):e70011. doi: 10.1111/sltb.70011 (PMC11959678; doi:10.1111/sltb.70011)
Supplement: Supplementary file 1 — Data S1. [file SLTB-55-0-s001.docx]

Supplemental Figure 1. Consort diagram for nocturnal wakefulness, cognitive control, and suicidal urges: A prospective evaluation of variance during residential treatment

­­ **Consort Diagram**

**Screened for Eligibility**

Number of charts reviewed (n= 314)

Number of charts potentially eligible for clinician contact (n= 222)

Not eligible (n= 92)

## Enrollment

**Result of Clinician Contact**

Number of veterans approved by clinicians (n=193)

Number of veterans disapproved by clinicians (n= 29)

High anxiety about suicide assessments upon admission (n = 1)

Issues w/ treatment focus and labile moods (n = 1)

No reason provided (n = 2)

Issues with treatment compliance (n = 1)

Not psychiatrically stable (n = 1)
 Psychotic symptoms (n = 1)

Knowledge of early discharge (n = 2)
 Behavioral issues (n = 1)

No response (n=19)

**Approached** (n= 145)

**Approached but not consented** (n = 70)

Plans to leave unit during study window (n=14)
 Noted they didn’t believe they would meet eligibility criteria/not interested (n=28)
 Number of veterans who left between approaching and consent (n=2)

Number of veterans who refused to consent (n= 26)

**Consented**

Number of veterans who consented (n= 75)

**Not approached** (n = 48)

Left prior to approach (n = 48)

**Eligibility**

Left unit prior to/during eligibility assessment (n= 6)
Excluded (n= 30)

Endorsed psychotic symptoms (n = 9)

Denied suicidal ideation (n = 18)
 Didn’t meet on ISI score (n = 3)

**Eligible (n = 39)**

Left unit or withdrew before enrollment (n = 3)

Consent was incomplete, no data included (n = 1)

**Enrolled (n = 35)**

Insufficient data collection for inclusion (n = 3)

Supplement 1

The interaction between period (between 7 AM – 11 PM) and nocturnal wakefulness was not significant (*F*(2,938)=0.86, *p*=0.42), indicating that time of day was not associated with S-VAS magnitude. The overall effect of nocturnal wakefulness, pooling over periods, trended towards significance (*F*(1,940)=3.67, *p*=0.055). The strength of this association was small to moderate; the (Spearman) correlation between nocturnal wakefulness and next-day S-VAS was 0.18 for the 7 AM-12 PM period, 0.21 for the 12 PM - 5 PM period, and 0.22 for the 5 PM-11 PM period. The significance of the nocturnal wakefulness effect on S-VAS was largely unchanged after controlling for age (*p* = .067), sex (*p* = .072), race (*p* = .064), while adjustment for baseline PTSD severity on the PTSD checklist (*p* = .029), baseline depression severity on the Beck Depression Inventory (*p* = .022), and baseline insomnia severity on the Insomnia Severity Index (*p* = .032) yielded significant adjusted effects.

| Supplemental Table 1  *Morning Sleep Diary Self-reported Time of Highest Suicidal Ideation Intensity on the Suicide-Visual Analogue Scale (S-VAS)* | | | | | | | | | | | | |
| --- | --- | --- | --- | --- | --- | --- | --- | --- | --- | --- | --- | --- |
| Hour | **ATUS**  **% Awake^1^** | **Scaled % Awake^2^** | **Expected # Awake^3^** | **Scaled Expected Awake^4^** | **Observed Frequency** | **Observed%^6^** | **Adjusted%^7^** | **Scaled%^8^** | **Standardized Incidence Ratio (SIR)^9^** | **95% CI for SIR (low)** | **95% CI for SIR (high)** | **SE** |
| 0:00 | 14.47% | 0.92% | 29.81 | 1.90 | 3 | 1.46 | 10.06 | 2.33 | 1.58 | 1.37 | 1.80 | 0.11 |
| 1:00 | 8.36% | 0.53% | 17.22 | 1.09 | 9 | 4.37 | 52.26 | 12.09 | **8.24** | 7.12 | 9.37 | 0.57 |
| 2:00 | 5.89% | 0.37% | 12.13 | 0.76 | 11 | 5.34 | 90.66 | 20.97 | **14.43** | 12.46 | 16.40 | 1.01 |
| 3:00 | 5.34% | 0.34% | 11.00 | 0.70 | 10 | 4.85 | 90.91 | 21.03 | **14.28** | 12.33 | 16.23 | 0.99 |
| 4:00 | 10.19% | 0.65% | 20.99 | 1.34 | 11 | 5.34 | 52.40 | 12.12 | **8.22** | 7.09 | 9.34 | 0.57 |
| 5:00 | 21.89% | 1.39% | 45.09 | 2.86 | 7 | 3.40 | 15.52 | 3.59 | **2.44** | 2.11 | 2.78 | 0.17 |
| 6:00 | 47.40% | 3.01% | 97.64 | 6.20 | 7 | 3.40 | 7.17 | 1.66 | 1.13 | 0.97 | 1.28 | 0.08 |
| 7:00 | 69.92% | 4.45% | 144.04 | 9.17 | 3 | 1.46 | 2.08 | 0.48 | **0.33** | 0.28 | 0.37 | 0.02 |
| 8:00 | 82.66% | 5.26% | 170.28 | 10.84 | 3 | 1.46 | 1.76 | 0.41 | **0.28** | 0.24 | 0.31 | 0.02 |
| 9:00 | 89.80% | 5.71% | 184.99 | 11.76 | 3 | 1.46 | 1.62 | 0.38 | **0.26** | 0.22 | 0.29 | 0.02 |
| 10:00 | 93.82% | 5.97% | 193.27 | 12.30 | 3 | 1.46 | 1.55 | 0.36 | **0.24** | 0.21 | 0.28 | 0.02 |
| 11:00 | 95.63% | 6.08% | 197.00 | 12.52 | 1 | 0.49 | 0.51 | 0.12 | **0.08** | 0.07 | 0.09 | 0.01 |
| 12:00 | 96.34% | 6.13% | 198.46 | 12.63 | 1 | 0.49 | 0.50 | 0.12 | **0.08** | 0.07 | 0.09 | 0.01 |
| 13:00 | 95.92% | 6.10% | 197.60 | 12.57 | 1 | 0.49 | 0.51 | 0.12 | **0.08** | 0.07 | 0.09 | 0.01 |
| 14:00 | 96.01% | 6.11% | 197.78 | 12.59 | 1 | 0.49 | 0.51 | 0.12 | **0.08** | 0.07 | 0.09 | 0.01 |
| 15:00 | 96.46% | 6.13% | 198.71 | 12.63 | 6 | 2.91 | 3.02 | 0.70 | **0.48** | 0.41 | 0.54 | 0.03 |
| 16:00 | 96.81% | 6.16% | 199.43 | 12.69 | 0 | 0.00 | 0.00 | 0.00 | **0.00** | 0.00 | 0.00 | 0.00 |
| 17:00 | 97.26% | 6.18% | 200.36 | 12.73 | 4 | 1.94 | 2.00 | 0.46 | **0.31** | 0.27 | 0.36 | 0.02 |
| 18:00 | 97.43% | 6.20% | 200.71 | 12.77 | 8 | 3.88 | 3.99 | 0.92 | **0.63** | 0.54 | 0.71 | 0.04 |
| 19:00 | 96.80% | 6.16% | 199.41 | 12.69 | 23 | 11.17 | 11.53 | 2.67 | 1.81 | 1.56 | 2.06 | 0.13 |
| 20:00 | 93.09% | 5.92% | 191.77 | 12.20 | 19 | 9.22 | 9.91 | 2.29 | 1.56 | 1.35 | 1.77 | 0.11 |
| 21:00 | 79.91% | 5.08% | 164.61 | 10.46 | 19 | 9.22 | 11.54 | 2.67 | 1.82 | 1.57 | 2.06 | 0.13 |
| 22:00 | 53.28% | 3.39% | 109.76 | 6.98 | 36 | 17.48 | 32.80 | 7.59 | **5.16** | 4.45 | 5.86 | 0.36 |
| 23:00 | 27.96% | 1.78% | 57.60 | 3.67 | 17 | 8.25 | 29.52 | 6.83 | **4.64** | 4.00 | 5.27 | 0.32 |
| SUM | 1572.64% | 100.00% | 3239.64 | 206.00 | 206 | 100.00 | 432.33 | 100.00 | -- | -- | -- | -- |
| Supplemental Table 1 Note: Bolded columns are significant. ^1^The proportion of the population awake at each hour based on ATUS; ^2^Proportions, divided by the total of all proportions (value from column 2, divided by the sum of all values from column 2). This scales each proportion so that they all sum to 100%; ^3^Values from column 2, multiplied by the total N=206. This represents the number of individuals from the sample expected to be awake at each hour; ^4^Values from column 4, divided by the total of column 4. This normalizes all values to a 100% scale; ^5^The observed number of suicides at each clock hour, from NVDRS; ^6^The proportion of suicides occurring at each clock hour compared to the entire sample (obtained by dividing each value from column 6 by the total N=206; ^7^The proportion of suicides occurring at each clock hour compared to the amount of the sample that is estimated to have been awake at that time (obtained by dividing the number of observed suicides from column 6 by the expected # awake in column 4); ^8^Values from column 8, divided by the sum of all values from column 8. This normalizes proportions so they all add up to 100; ^9^SIR computed by dividing the observed frequency from column 6 (i.e., observed) by the scaled number expected awake from column 5 (i.e., expected); ^10^95% Confidence Interval (95%CI) computed around the SIR; ^11^P value for SIR | | | | | | | | | | | | |

|  |
| --- |
|  |
|  |
|  |
|  |
|  |
|  |
|  |
|  |
| \| Supplemental Table 2  *Evening Sleep Diary Self-reported Time of Highest Suicidal Ideation Intensity on the Suicide-Visual Analogue Scale (S-VAS)* \| \| \| \| \| \| \| \| \| \| \| \| \| \| --- \| --- \| --- \| --- \| --- \| --- \| --- \| --- \| --- \| --- \| --- \| --- \| --- \| \| Hour \| **ATUS**  **% Awake^1^** \| **Scaled % Awake^2^** \| **Expected # Awake^3^** \| **Scaled Expected Awake^4^** \| **Observed Frequency** \| **Observed%^6^** \| **Adjusted%^7^** \| **Scaled%^8^** \| **Standardized Incidence Ratio (SIR)^9^** \| **95% CI for SIR (low)** \| **95% CI for SIR (high)** \| **SE** \| \| 0:00 \| 14.47% \| 0.92% \| 21.27 \| 1.35 \| 0 \| 0.00 \| 0.00 \| 0.00 \| 0.00 \| 0.00 \| 0.00 \| 0.00 \| \| 1:00 \| 8.36% \| 0.53% \| 12.29 \| 0.78 \| 0 \| 0.00 \| 0.00 \| 0.00 \| 0.00 \| 0.00 \| 0.00 \| 0.00 \| \| 2:00 \| 5.89% \| 0.37% \| 8.66 \| 0.54 \| 0 \| 0.00 \| 0.00 \| 0.00 \| 0.00 \| 0.00 \| 0.00 \| 0.00 \| \| 3:00 \| 5.34% \| 0.34% \| 7.85 \| 0.50 \| 0 \| 0.00 \| 0.00 \| 0.00 \| 0.00 \| 0.00 \| 0.00 \| 0.00 \| \| 4:00 \| 10.19% \| 0.65% \| 14.98 \| 0.96 \| 3 \| 2.04 \| 20.03 \| 4.63 \| **3.14** \| 2.63 \| 3.65 \| 0.26 \| \| 5:00 \| 21.89% \| 1.39% \| 32.18 \| 2.04 \| 4 \| 2.72 \| 12.43 \| 2.88 \| 1.96 \| 1.64 \| 2.27 \| 0.16 \| \| 6:00 \| 47.40% \| 3.01% \| 69.68 \| 4.42 \| 8 \| 5.44 \| 11.48 \| 2.66 \| 1.81 \| 1.52 \| 2.10 \| 0.15 \| \| 7:00 \| 69.92% \| 4.45% \| 102.78 \| 6.54 \| 3 \| 2.04 \| 2.92 \| 0.68 \| **0.46** \| 0.38 \| 0.53 \| 0.04 \| \| 8:00 \| 82.66% \| 5.26% \| 121.51 \| 7.73 \| 5 \| 3.40 \| 4.11 \| 0.95 \| **0.65** \| 0.54 \| 0.75 \| 0.05 \| \| 9:00 \| 89.80% \| 5.71% \| 132.01 \| 8.39 \| 5 \| 3.40 \| 3.79 \| 0.88 \| **0.60** \| 0.50 \| 0.69 \| 0.05 \| \| 10:00 \| 93.82% \| 5.97% \| 137.92 \| 8.78 \| 4 \| 2.72 \| 2.90 \| 0.67 \| **0.46** \| 0.38 \| 0.53 \| 0.04 \| \| 11:00 \| 95.63% \| 6.08% \| 140.58 \| 8.94 \| 5 \| 3.40 \| 3.56 \| 0.82 \| **0.56** \| 0.47 \| 0.65 \| 0.05 \| \| 12:00 \| 96.34% \| 6.13% \| 141.62 \| 9.01 \| 8 \| 5.44 \| 5.65 \| 1.31 \| 0.89 \| 0.74 \| 1.03 \| 0.07 \| \| 13:00 \| 95.92% \| 6.10% \| 141.00 \| 8.97 \| 7 \| 4.76 \| 4.96 \| 1.15 \| **0.78** \| 0.65 \| 0.91 \| 0.06 \| \| 14:00 \| 96.01% \| 6.11% \| 141.13 \| 8.98 \| 7 \| 4.76 \| 4.96 \| 1.15 \| **0.78** \| 0.65 \| 0.91 \| 0.06 \| \| 15:00 \| 96.46% \| 6.13% \| 141.80 \| 9.01 \| 12 \| 8.16 \| 8.46 \| 1.96 \| 1.33 \| 1.12 \| 1.55 \| 0.11 \| \| 16:00 \| 96.81% \| 6.16% \| 142.31 \| 9.06 \| 6 \| 4.08 \| 4.22 \| 0.98 \| **0.66** \| 0.56 \| 0.77 \| 0.05 \| \| 17:00 \| 97.26% \| 6.18% \| 142.97 \| 9.08 \| 4 \| 2.72 \| 2.80 \| 0.65 \| **0.44** \| 0.37 \| 0.51 \| 0.04 \| \| 18:00 \| 97.43% \| 6.20% \| 143.22 \| 9.11 \| 3 \| 2.04 \| 2.09 \| 0.48 \| **0.33** \| 0.28 \| 0.38 \| 0.03 \| \| 19:00 \| 96.80% \| 6.16% \| 142.30 \| 9.06 \| 22 \| 14.97 \| 15.46 \| 3.58 \| **2.43** \| 2.04 \| 2.82 \| 0.20 \| \| 20:00 \| 93.09% \| 5.92% \| 136.84 \| 8.70 \| 15 \| 10.20 \| 10.96 \| 2.54 \| 1.72 \| 1.45 \| 2.00 \| 0.14 \| \| 21:00 \| 79.91% \| 5.08% \| 117.47 \| 7.47 \| 16 \| 10.88 \| 13.62 \| 3.15 \| 2.14 \| 1.80 \| 2.49 \| 0.18 \| \| 22:00 \| 53.28% \| 3.39% \| 78.32 \| 4.98 \| 8 \| 5.44 \| 10.21 \| 2.36 \| 1.61 \| 1.35 \| 1.86 \| 0.13 \| \| 23:00 \| 27.96% \| 1.78% \| 41.10 \| 2.62 \| 2 \| 1.36 \| 4.87 \| 1.13 \| **0.76** \| 0.64 \| 0.89 \| 0.06 \| \| SUM \| 1572.64% \| 100.00% \| 2311.78 \| 147 \| 147 \| 100.00 \| 149.49 \| 34.58 \| -- \| -- \| -- \| -- \| \| Supplemental Table 1 Note: Bolded columns are significant. ^1^The proportion of the population awake at each hour based on ATUS; ^2^Proportions, divided by the total of all proportions (value from column 2, divided by the sum of all values from column 2). This scales each proportion so that they all sum to 100%; ^3^Values from column 2, multiplied by the total N=147. This represents the number of individuals from the sample expected to be awake at each hour; ^4^Values from column 4, divided by the total of column 4. This normalizes all values to a 100% scale; ^5^The observed number of suicides at each clock hour, from NVDRS; ^6^The proportion of suicides occurring at each clock hour compared to the entire sample (obtained by dividing each value from column 6 by the total N=147; ^7^The proportion of suicides occurring at each clock hour compared to the amount of the sample that is estimated to have been awake at that time (obtained by dividing the number of observed suicides from column 6 by the expected # awake in column 4); ^8^Values from column 8, divided by the sum of all values from column 8. This normalizes proportions so they all add up to 100; ^9^SIR computed by dividing the observed frequency from column 6 (i.e., observed) by the scaled number expected awake from column 5 (i.e., expected); ^10^95% Confidence Interval (95%CI) computed around the SIR; ^11^P value for SIR \| \| \| \| \| \| \| \| \| \| \| \| \| |
|  |
|  |

| Supplemental Table 3  *Combined Morning and Evening Sleep Diary Self-reported Time of Highest Suicidal Ideation Intensity on the Suicide-Visual Analogue Scale (S-VAS)* | | | | | | | | | | | | |
| --- | --- | --- | --- | --- | --- | --- | --- | --- | --- | --- | --- | --- |
| Hour | **ATUS**  **% Awake^1^** | **Scaled % Awake^2^** | **Expected # Awake^3^** | **Scaled Expected Awake^4^** | **Observed Frequency** | **Observed%^6^** | **Adjusted%^7^** | **Scaled%^8^** | **Standardized Incidence Ratio (SIR)^9^** | **95% CI for SIR (low)** | **95% CI for SIR (high)** | **SE** |
| 0:00 | 14.47% | 0.92% | 51.08 | 3.25 | 3 | 0.85 | 5.87 | 1.36 | 0.92 | 0.83 | 1.02 | 0.05 |
| 1:00 | 8.36% | 0.53% | 29.51 | 1.87 | 9 | 2.55 | 30.50 | 7.05 | **4.81** | 4.03 | 5.59 | 0.40 |
| 2:00 | 5.89% | 0.37% | 20.79 | 1.31 | 11 | 3.12 | 52.91 | 12.24 | **8.42** | 7.06 | 9.78 | 0.69 |
| 3:00 | 5.34% | 0.34% | 18.85 | 1.20 | 10 | 2.83 | 53.05 | 12.27 | **8.33** | 6.99 | 9.68 | 0.69 |
| 4:00 | 10.19% | 0.65% | 35.97 | 2.29 | 14 | 3.97 | 38.92 | 9.00 | **6.10** | 5.12 | 7.09 | 0.50 |
| 5:00 | 21.89% | 1.39% | 77.27 | 4.91 | 11 | 3.12 | 14.24 | 3.29 | 2.24 | 1.88 | 2.60 | 0.18 |
| 6:00 | 47.40% | 3.01% | 167.32 | 10.63 | 15 | 4.25 | 8.96 | 2.07 | 1.41 | 1.18 | 1.64 | 0.12 |
| 7:00 | 69.92% | 4.45% | 246.82 | 15.71 | 6 | 1.70 | 2.43 | 0.56 | **0.38** | 0.32 | 0.44 | 0.03 |
| 8:00 | 82.66% | 5.26% | 291.79 | 18.57 | 8 | 2.27 | 2.74 | 0.63 | **0.43** | 0.36 | 0.50 | 0.04 |
| 9:00 | 89.80% | 5.71% | 316.99 | 20.16 | 8 | 2.27 | 2.52 | 0.58 | **0.40** | 0.33 | 0.46 | 0.03 |
| 10:00 | 93.82% | 5.97% | 331.18 | 21.07 | 7 | 1.98 | 2.11 | 0.49 | **0.33** | 0.28 | 0.39 | 0.03 |
| 11:00 | 95.63% | 6.08% | 337.57 | 21.46 | 6 | 1.70 | 1.78 | 0.41 | **0.28** | 0.23 | 0.32 | 0.02 |
| 12:00 | 96.34% | 6.13% | 340.08 | 21.64 | 9 | 2.55 | 2.65 | 0.61 | **0.42** | 0.35 | 0.48 | 0.03 |
| 13:00 | 95.92% | 6.10% | 338.60 | 21.53 | 8 | 2.27 | 2.36 | 0.55 | **0.37** | 0.31 | 0.43 | 0.03 |
| 14:00 | 96.01% | 6.11% | 338.92 | 21.57 | 8 | 2.27 | 2.36 | 0.55 | **0.37** | 0.31 | 0.43 | 0.03 |
| 15:00 | 96.46% | 6.13% | 340.50 | 21.64 | 18 | 5.10 | 5.29 | 1.22 | **0.83** | 0.70 | 0.97 | 0.07 |
| 16:00 | 96.81% | 6.16% | 341.74 | 21.74 | 6 | 1.70 | 1.76 | 0.41 | **0.28** | 0.23 | 0.32 | 0.02 |
| 17:00 | 97.26% | 6.18% | 343.33 | 21.82 | 8 | 2.27 | 2.33 | 0.54 | **0.37** | 0.31 | 0.43 | 0.03 |
| 18:00 | 97.43% | 6.20% | 343.93 | 21.89 | 11 | 3.12 | 3.20 | 0.74 | **0.50** | 0.42 | 0.58 | 0.04 |
| 19:00 | 96.80% | 6.16% | 341.70 | 21.74 | 45 | 12.75 | 13.17 | 3.05 | 2.07 | 1.73 | 2.40 | 0.17 |
| 20:00 | 93.09% | 5.92% | 328.61 | 20.90 | 34 | 9.63 | 10.35 | 2.39 | 1.63 | 1.36 | 1.89 | 0.13 |
| 21:00 | 79.91% | 5.08% | 282.08 | 17.93 | 35 | 9.92 | 12.41 | 2.87 | 1.95 | 1.64 | 2.27 | 0.16 |
| 22:00 | 53.28% | 3.39% | 188.08 | 11.97 | 44 | 12.46 | 23.39 | 5.41 | **3.68** | 3.08 | 4.27 | 0.30 |
| 23:00 | 27.96% | 1.78% | 98.70 | 6.28 | 19 | 5.38 | 19.25 | 4.45 | **3.02** | 2.54 | 3.51 | 0.25 |
| SUM | 1572.64% | 100.00% | 5551.42 | 353 | 353 | 100.00 | 314.54 | 72.76 | -- | -- | -- | -- |
| Supplemental Table 1 Note: Bolded columns are significant. ^1^The proportion of the population awake at each hour based on ATUS; ^2^Proportions, divided by the total of all proportions (value from column 2, divided by the sum of all values from column 2). This scales each proportion so that they all sum to 100%; ^3^Values from column 2, multiplied by the total N=353. This represents the number of individuals from the sample expected to be awake at each hour; ^4^Values from column 4, divided by the total of column 4. This normalizes all values to a 100% scale; ^5^The observed number of suicides at each clock hour, from NVDRS; ^6^The proportion of suicides occurring at each clock hour compared to the entire sample (obtained by dividing each value from column 6 by the total N=353; ^7^The proportion of suicides occurring at each clock hour compared to the amount of the sample that is estimated to have been awake at that time (obtained by dividing the number of observed suicides from column 6 by the expected # awake in column 4); ^8^Values from column 8, divided by the sum of all values from column 8. This normalizes proportions so they all add up to 100; ^9^SIR computed by dividing the observed frequency from column 6 (i.e., observed) by the scaled number expected awake from column 5 (i.e., expected); ^10^95% Confidence Interval (95%CI) computed around the SIR; ^11^P value for SIR | | | | | | | | | | | | |
